# A novel predictor of mortality for penetrating brain injury in paediatric patients

**DOI:** 10.12669/pjms.41.12.12172

**Published:** 2025-12

**Authors:** Mustafa Bolatkale, Kursat Kaan Kerimoglu, Yahya Efe Guner, Ahmet Cagdas Acara

**Affiliations:** 1 Mustafa Bolatkale, MD, Associate Professor, Department of Emergency Medicine, Alsancak Nevvar-Salih Isgoren State Hospital, Konak, Izmir, Turkiye; 2 Kursat Kaan Kerimoglu, MD, Department of Emergency Medicine, Alsancak Nevvar-Salih Isgoren State Hospital, Konak, Izmir, Turkiye; 3 Yahya Efe Guner, MD, Department of Anatomy, Ankara University, Graduate School of Health Sciences, Ankara, Turkiye; 4 Ahmet Cagdas Acara, MD, Clinics of Emergency Medicine, Health Science University, Izmir Bozyaka Training and Research Hospital, Karabaglar, Izmir, Turkiye

**Keywords:** Computed tomography, Foreign body, Penetrating brain injury, Paediatric neurotrauma, Sniper, Shrapnel

## Abstract

**Objective:**

Penetrating brain injury (PBI) is a severe form of traumatic brain injury associated with high mortality, particularly in children. However, mortality predictors in children with PBI remain unclear. This study aimed to evaluate the association among the number of intracranial foreign bodies (FBs), brain computed tomography (CT) findings, and mortality outcomes in paediatric patients with PBI who presented to the emergency department (ED).

**Methodology:**

This prospective observational study was conducted in the ED of Kilis State Hospital from December 2015 to December 2022. Paediatric patients with isolated penetrating head injuries from sniper gunshot wounds or barrel bomb explosions were included. Demographic data, pre-hospital interventions, Glasgow Coma Scale scores, brain CT findings, and mortality outcomes were collected and analysed.

**Results:**

This study included 126 paediatric patients with PBI. The presence of ≥2 intracranial FBs was significantly associated with increased mortality (odds ratio 37.4; p<0.001). Other mortality predictors included Glasgow Coma Scale score ≤9, epidural haematoma, midline shift, and pre-hospital endotracheal intubation. Mortality was not significantly different between injuries from shrapnel and those from sniper gunshots.

**Conclusion:**

In paediatric patients with PBI, having ≥2 intracranial FBs was associated with increased mortality. Therefore, FB count may be a novel and accessible prognostic factor. Further research is required to validate these findings and support their integration into clinical decision-making.

## INTRODUCTION

Traumatic brain injury (TBI) significantly contributes to deaths and long-term disabilities in children and adolescents in the United States.^1^,^2^ Annually, approximately 1.7 million cases are reported, resulting in 275,000 hospitalisations, 52,000 fatalities, and an economic impact of $76 billion.^3^ Penetrating brain injury (PBI), a distinct form of TBI, results from non-blunt mechanisms and involves skull penetration with direct brain tissue damage.^4^,^5^ Despite treatment improvements, 70-90% of patients with PBI die before reaching the hospital; almost half of those who make it to the emergency department (ED) do not survive resuscitation.^5^ PBI carries a mortality rate of 23-93% and often results in permanent neurological deficits.^6^

In regions experiencing armed conflict, bomb explosions and gunfire frequently result in injuries impacting military personnel and civilians.^7^ The incidence of war-related PBI has increased, particularly in conflicts such as those in Syria, Afghanistan, and Iraq, where gunshot wounds and barrel bomb explosions have caused numerous severe injuries.^8^ Prompt and appropriate treatment of patients with PBI may enhance outcomes.^9^ Computed tomography (CT) is crucial in detecting cerebrovascular injuries following PBI by identifying foreign bodies (FBs), assessing lesion extent, localising fragments, detecting hematomas, determining penetration pathways, and guiding surgical intervention planning.^10^

Timely and high-quality acute care is essential in managing patients with PBI.^11^ The ED is the primary site for initial PBI management and data collection; improved acute care, specialised protocols, and urgent surgical interventions enhance outcomes in children with severe injuries, highlighting the need for neurosurgical expertise in conflict zones.^12^,^13^ PBI management is challenging for emergency physicians and neurosurgeons, as timely surgical intervention is critical to reducing mortality and minimising complications.^14^ Mortality is influenced by various factors, including the Glasgow Coma Scale (GCS) score, coagulopathy, hypotension, patient age, clinical signs indicative of elevated intracranial pressure, and CT findings that encompass various types of haemorrhages, herniation, pneumocephalus, hydrocephalus, and calvarial fractures.^15^,^16^

The presence of multiple intracranial FBs significantly increases the mortality risk among adults.^17^ However, available evidence concerning paediatric PBI cases, particularly those from sniper gunshot wounds and barrel bomb explosions, remains limited. Further research is imperative to ascertain mortality predictors and enhance outcomes in paediatric populations.

This study investigated the association between the number of intracranial FBs and mortality in paediatric patients with PBI from sniper gunshot wounds or barrel bomb explosions. We also assessed the correlation between brain CT findings and mortality outcomes in this population.

## METHODOLOGY

This prospective observational study was conducted in the ED of Kilis State Hospital, a rural hospital near the Syrian border. The study spanned seven years (December 23, 2015 to December 23, 2022). During the study period, 4,317 patients were admitted to the ED with polytrauma resulting from terrorist attacks during the Syrian Civil War (SCW). After the exclusion criteria were applied, 126 paediatric patients with isolated penetrating head injuries were identified: 57 sustained injuries from barrel bomb explosions, and 69 were affected by sniper gunshot wounds. The study population comprised 126 patients.

### Ethical approval:

This study was approved by the Clinical Research Ethics Committee of Medipol University (approval date: December 23, 2015; protocol number: E.5143-647).

### Inclusion and Exclusion criteria:

Paediatric patients with PBI from sniper gunshot wounds or barrel bomb explosions demonstrating evidence of intracranial metallic FBs on CT were included. The exclusion criteria were injuries to body parts other than the head, low-velocity gunshot wounds, tangential head injuries, absence of metallic fragments on CT, and fatalities on the battlefield or within 24 hours of transfer.

### Data collection:

Demographic data (age and sex), pre-hospital interventions (including pre-hospital endotracheal intubation), initial GCS scores, and mortality outcomes were recorded. Brain CT scans were reviewed to assess the number of intracranial metallic FBs and identify associated findings, such as epidural haematoma (EDH), subarachnoid haemorrhage (SAH), subdural haematoma, intracerebral haemorrhage (ICH), pneumocephalus, and midline shift. Additionally, the anatomical locations of skull fractures were documented. All CT images were independently evaluated by two neurosurgeons who had experienced paediatric neurotrauma. In cases of discrepancy, the findings were reviewed jointly, and a consensus was reached to confirm the number and localisation of intracranial FBs.

### Statistical analysis:

MedCalc software 14 (Acacialaan 22, B-8400 Ostend, Belgium) and Statistical Package for the Social Sciences for Windows, version 22.0 (IBM Corporation, Armonk, NY, USA) were used for statistical analysis. Normality of the distribution of continuous variables was assessed using the Shapiro-Wilk test. Variance homogeneity was evaluated using Levene’s test. The Mann-Whitney U test, in conjunction with Monte Carlo results, was used to compare continuous variables between two independent groups. Pearson’s chi-square test and Fisher’s exact test were used with Monte Carlo simulation to compare categorical variables. Receiver operating characteristic (ROC) curve analysis was performed to determine the optimal cut-off value of FB count for predicting mortality. Multiple logistic regression analysis, employing the backward selection method, was used to determine cause-and-effect relationships between categorical and explanatory variables. Quantitative variables are presented as means (standard deviations) and medians (ranges), while categorical variables are presented as frequencies (percentages). All analyses were conducted at a 95% confidence level, and statistical significance was set at p<0.05.

## RESULTS

During the study, 4,317 patients were admitted to the ED with polytrauma related to terrorist attacks from the SCW. After applying the exclusion criteria, we identified 126 paediatric patients with isolated head injuries: 57 due to bomb explosions and 69 due to sniper gunshot wounds. The study population consisted of 126 patients (89.7% male and 10.3% female; mean age 10.7 years). Those who survived the first 24 hour of admission and those who did not survive in the ED were compared. The groups were named ‘responsive to treatment’ (n=59) and ‘non-responsive to treatment’ (n=67). Factors affecting mortality were compared between these groups. We examined the impact of intracranial FB count on mortality and determined an optimal cut-off value for mortality by performing ROC analysis of intracranial FB count in our study population. We also assessed the correlation between the number of intracranial FBs and mortality. The ROC curve analysis indicated that the optimal cut-off value for predicting mortality was ≥2 FBs, based on which the total study population was divided into ‘FB <2’ (n=69) and ‘FB ≥2’ (n=57) groups. The variables were compared between these groups. Subsequent analyses compared the PBI from sniper gunshots (sniper group, n=69) with that from barrel bomb explosion shrapnel (shrapnel group, n=57). Factors affecting mortality were analysed between these groups.

In total, 126 patients received treatment in the ED, which included correcting hypotension and hypoxia, maintaining airway patency, managing any associated haemorrhage, administering hyperosmolar therapy with mannitol or hypertonic saline, correcting traumatic coagulopathy, applying cervical immobilisation devices, and providing tetanus and antibiotic prophylaxis. Upon arrival at the ED, all patients were evaluated by a neurosurgeon. Despite receiving treatment within 24 hours of admission to the ED, 67 of 126 patients (53.17%) died; the remaining 59 patients were admitted for surgery. Among them, 36 underwent decompressive surgery and 23 underwent craniotomy.

**Fig.1:**
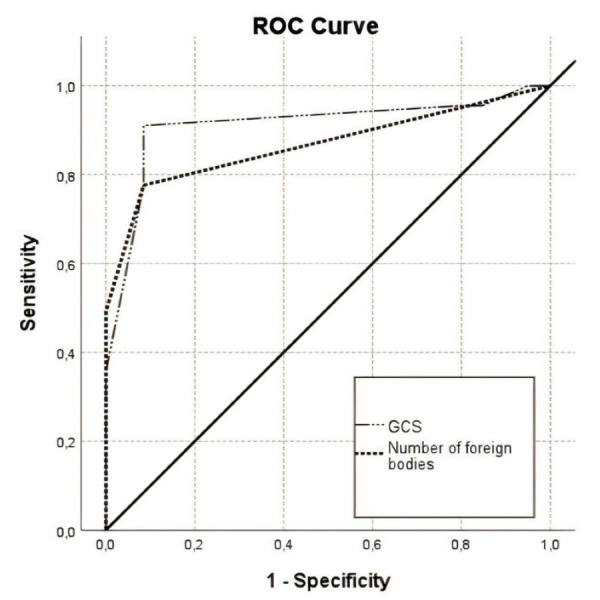
Receiver operating characteristic curves of the number of Foreign Bodies and Glasgow Coma Scale (cut-off points are 2 and 9, respectively).

Temporal and occipital bone fractures, ICH, SAH, EDH, pneumocephalus, midline shift, transventricular injuries, pre-hospital endotracheal intubation (PEI), two-lobe injuries, and brainstem involvement were associated with higher mortality rates. The treatment-responsive group showed a significantly higher rate of GCS scores >9 and one-lobe injuries (t-test, p<0.05) (Table-I). ICH (p<0.032), frontal bone fracture (p<0.007), parietal bone fracture (p<0.013), number of FBs (p=0.009), and pneumocephalus (p=0.039) were significantly different between the sniper and shrapnel groups, with a higher mortality observed in the shrapnel group (p<0.05) (Table-I). On ROC curve analysis, FB count ≥2 showed 77.6% sensitivity, 91.5% specificity, 78.3% positive predictive value (PPV), and 91.2% negative predictive value (NPV) for predicting mortality (area under the curve: 0.867±0.034; p<0.001) (Fig.1).

**Table-I:** Comparison of clinical and radiological characteristics among different patient groups.

Variables	Treatment Response Groups				Sniper/Shrapnel Groups			FB < 2 and FB ≥ 2 groups		
	Responsive (n = 59)	Non-Responsive (n = 67)	P value	Sniper (n = 69)		Shrapnel (n = 57)	P value	Number of FBs < 2 (n = 69)	Number of FBs ≥ 2 (n = 57)	P value
Age, med(min/max)	11 (1 / 16)	12 (2 / 17)	0.859 ᵘ	12 (1 / 17)		9 (4 / 17)	0.059 ᵘ	12 (1 / 16)	11 (3 / 17)	0.877 ᵘ
Gender, n (%)	47 (79.7)	66 (98.5)	*0.001* ᶜ	62 (89.9)		51 (89.5)	0.999 ᶜ	57 (82.6)	56 (98.2)	*0.006 ᶠ*
SDH, n (%)	3 (5.1)	9 (13.4)	0.136 ᶜ	6 (8.7)		6 (10.5)	0.768 ᶜ	6 (8.7)	6 (10.5)	0.728 c
EDH, n (%)	12 (20.3)	21 (31.3)	0.223 ᶜ	15 (21.7)		18 (31.6)	0.228 ᶜ	9 (13.0)	24 (42.1)	*<0.001* ᶜ
ICH, n (%)	53 (89.8)	67 (100)	*0.009* ᶠ	63 (91.3)		57 (100)	*0.032* ᶠ	63 (91.3)	57 (100.0)	*0.032* ᶠ
SAH, n (%)	6 (10.2)	21 (31.3)	*0.005* ᶜ	12 (17.4)		15 (26.3)	0.277 ᶜ	15 (21.7)	12 (21.1)	0.926 ᶜ
Frontal, n (%)	33 (55.9)	36 (53.7)	0.859 ᶜ	30 (43.5)		39 (68.4)	*0.007* ᶜ	36 (52.2)	33 (57.9)	0.521 ᶜ
Parietal, n (%)	38 (64.4)	46 (68.7)	0.706 ᶜ	39 (56.5)		45 (78.9)	*0.013* ᶜ	36 (52.2)	48 (85.2)	*<0.001* ᶜ
Temporal, n (%)	20 (33.9)	49 (73.1)	*<0.001* ᶜ	33 (47.8)		36 (63.2)	0.106 ᶜ	30 (43.5)	39 (68.4)	*0.005* ᶜ
Occipital, n (%)	6 (10.2)	27 (40.3)	*<0.001* ᶜ	18 (26.1)		15 (26.3)	0.999 ᶜ	12 (17.4)	21 (36.8)	*0.013* ᶜ
Number of FB, med (min/max)	1 (1 / 2)	2 (1 / 9)	*<0.001* ᵘ	31 (44.9)		36 (63.2)	*0.049* ᶜ	15 (21.7)	52 (91.2)	*<0.001* ᶜ
GCS, med (min/max)	10 (8 / 13)	8 (3 / 12)	*<0.001* ᵘ	1 (1 / 4)		2 (1 / 9)	*0.009* ᵘ	10 (8 / 13)	8 (3 / 12)	*<0.001* ᵘ
Sniper, n (%)	38 (64.4)	25 (37.3)	*0.004* ᶜ	10 (3 / 13)		8 (3 / 12)	*0.035* ᵘ	42 (60.9)	21 (36.8)	*0.007* ᶜ
Shrapnel, n (%)	21 (35.6)	42 (62.7)	*0.004* ᶜ	21 (30.4)		13 (22.8)	0.421 ᶜ	27 (39.1)	36 (63.2)	*0.007* ᶜ
Injury Type, n (%)	21 (35.6)	36 (53.7)	*0.049* ᶜ	21 (30.4)		18 (31.6)	0.999 ᶜ	27 (39.1)	30 (52.6)	0.130 ᶜ
PEI, n (%)	9 (15.3)	25 (37.3)	*0.008* ᶜ	51 (73.9)		51 (89.5)	*0.039* ᶜ	7 (10.1)	27 (47.4)	*<0.001* ᶜ
Midline shift, n (%)	11 (18.6)	28 (41.8)	*0.007* ᶜ	33 (47.8)		24 (42.1)	0.591 ᶜ	12 (17.4)	27 (47.4)	*<0.001* ᶜ
Pneumocephalus, n (%)	35 (59.3)	67 (100)	*<0.001* ᶜ	36 (52.2)		33 (57.9)	0.591 ᶜ	45 (65.2)	57 (100.0)	*<0.001* ᶜ
One lobe, n (%)	39 (66.1)	18 (26.9)	*<0.001* ᶜ	21 (30.4)		24 (42.1)	0.195 ᶜ	42 (60.9)	15 (26.3)	*<0.001* ᶜ
Two lobes, n (%)	20 (33.9)	49 (73.1)	*<0.001* ᶜ	12 (17.4)		15 (26.3)	0.277 ᶜ	27 (39.1)	42 (73.7)	*<0.001* ᶜ
Trans-ventricular, n (%)	14 (23.7)	31 (46.3)	*0.010* ᶜ	63 (91.3)		45 (78.9)	0.072 ᶜ	18 (26.1)	27 (47.4)	*0.013* ᶜ
Brainstem involvement, n (%)	6 (10.2)	21 (31.3)	*0.005* ᶜ	6 (8.7)		12 (21.1)	0.072 ᶜ	9 (13.0)	18 (31.6)	*0.012* ᶜ
Penetrating, n (%)	50 (84.7)	58 (86.6)	0.803 ᶜ	12 (1 / 17)		9 (4 / 17)	0.059 ᵘ	57 (82.6)	51 (89.5)	0.273 c
Perforating, n (%)	9 (15.3)	9 (13.4)	0.803 ᶜ	62 (89.9)		51 (89.5)	0.999 ᶜ	12 (17.4)	6 (10.5)	0.273 ᶠ

ᵘ Mann Whitney U Test (Monte Carlo), ᶜ Pearson Chi Square Test (Monte Carlo), ᶠ Fisher Exact Test (Monte Carlo), min: Minimum, max: Maximum, FB: Foreign body, med: Median, SDH: Subdural hematoma, EDH: Epidural hematoma, ICH: Intracerebral hemorrhage, SAH: Subarachnoid hemorrhage, GCS: Glasgow Coma Scale, PEI: Prehospital endotracheal intubation.

Parietal, temporal, and occipital bone fractures, ICH, pneumocephalus, two-lobe injuries, transventricular injuries, and brainstem involvement were significantly more frequent in the FB ≥2 group (p<0.05) (Table-I). Conversely, one-lobe injuries and GCS scores >9 were significantly more prevalent in the FB <2 group (p<0.05) (Table-I). Patients with ≥2 intracranial FBs had a 37.4-fold higher mortality rate than those with <2 FBs (odds ratio [OR]: 37.4, 95% confidence interval [CI] 12.6-110.4, p<0.001) (Table-II).

**Table-II:** ROC analysis for Glasgow Coma Scale and the number of foreign bodies.

		Responsive to Treatment (n = 59)			Non-responsive to treatment (n = 67)			AUC ± SE	Odds Ratio (95% CI)	P value
		n	Row N (%)	Column N (%)	n	Row N (%)	Column N (%)			
**GCS**	>9	54	90.0^3^	91.5^2^	6	10.0	9.0	0.911 ± 0.029	109.8 (31.7 - 380.1)	*< .001*
	≤9	5	7.6	8.5	61	92.4^4^	91.0^1^			
**Number of FBs**	<2	54	78.3^3^	91.5^2^	15	21.7	22.4	0.867 ± 0.034	37.4(12.6 - 110.4)	*< .001*
	≥2	5	8.8	8.5	52	91.2^4^	77.6^1^			

ROC: Receiver Operating Curve (Honley & Mc Nell - Youden index J), AUC: Area under the ROC curve, GCS: Glasgow Coma Scale, FB: Foreign body, SE: Standard error, CI: Confidence interval.

1 Sensitivity,

2 Specificity,

3 Positive predictive value,

4 Negative predictive value

All variables were analysed to identify factors associated with FB count. After confounding variables were accounted for, GCS score (p<0.001), EDH (p<0.001), PEI (p<0.001), and midline shift (p=0.003) were significantly associated with FB count (Table-III). The optimal cut-off GCS score for predicting mortality was ≤9, exhibiting a 91.0% sensitivity, 91.5% specificity, 90.0% PPV, and 92.4% NPV (area under the curve: 0.911±0.029; p<0.001) (Fig.1, Table-IV). Patients with GCS ≤9 exhibited a 109.8-fold higher mortality rate than those with GCS <9 (OR 109.8, 95% CI 31.7-380.1, p<0.001) (Table-II).

**Table-III:** The number of FBs, GCS, EDH, PEI and midline shift of multiple logistic regression analysis.

Reference Groups: Number of FBs ≥ 2	B (SE.)	P Value	Odds Ratio (95% C.I.)
GCS	4.44 (0.67)	*<0.001*	85.50 [22.86 - 319.66]
EDH	2.47 (0.65)	*<0.001*	11.87 [3.30 - 42.74]
PEI	1.82 (0.55)	*<0.001*	6.22 [2.09 - 18.44]
Midline shift	1.81 (0.61)	*0,003*	6.16 [1.84 - 20.63]
Constant	2.14 (0.48)	*<0.001*	-
Accuracy rates	Number of FBs < 2: 87.0	Number of FBs **≥** 2: 73.7	General: 81.0

Multiple Logistic Regression (Method = Backward Stepwise (Wald)), C.I.: Confidence interval B: regression coefficients SE.: Standard Error, ᴿ Reference Group, GCS: Glasgow Coma Scale, FB: Foreign body, EDH: Epidural hematoma, PEI: Prehospital endotracheal intubation.

**Fig.2:**
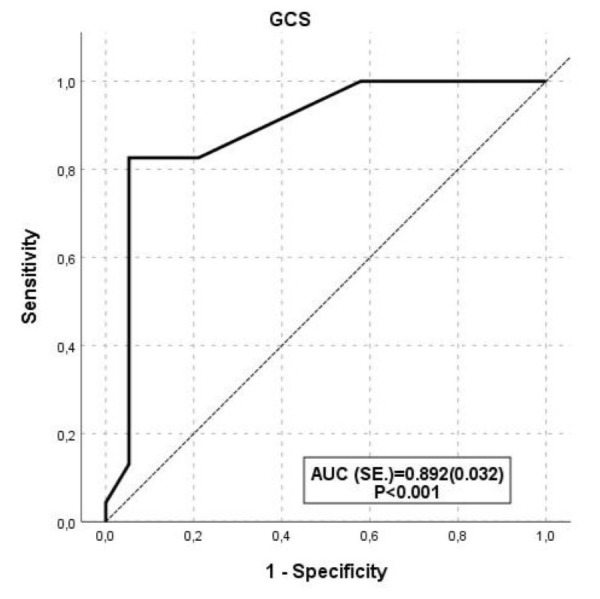
Receiver operating characteristic curve of Glasgow Coma Scale according to number of Foreign Bodies (optimal cut-off value is 9).

A significant association was observed between GCS scores and FB count <2, showing a 16.3-fold increase (OR 16.3, 95% CI 5.39-49.59) (p<0.001); the optimal cut-off value for GCS was determined to be ≤9 based on intracranial FB count (p<0.001) (Table-IV, Fig.2). Furthermore, a significant negative correlation was observed between GCS scores and FB count (r=-0.789, p<0.001).

**Table-IV:** Receiver operating characteristic analysis for Glasgow Coma Scale according to number of Foreign Bodies.

References: Number of FBs ≥ 2	Cut off	Sensitivity	Specificity	+PV	-PV	AUC ± SE.	OR (95% CI)	P Value
**GCS**	≤ 9	81.8	95.0	82.6	94.7	0.892 ± 0.032	16.3 (5.39-49.59)	*<0.001*

ROC (Receiver Operating Curve) Analysis (Honley&Mc Nell - Youden index J), AUC: Area under the ROC curve, SE: Standard Error, +PV: Positive Predictive Value, -PV: Negative Predictive Value, GCS: Glasgow Coma Scale, FB: Foreign body.

Patients with ≥2 FBs had an 11.87-fold higher risk of EDH (OR 11.87, 95% CI 3.30-42.74, p<0.001), a 6.16-fold higher risk of midline shift (OR 6.16, 95% CI 1.84-20.63, p=0.003), and a 6.22-fold higher risk of PEI (OR 6.22, 95% CI 2.09-18.44) (p<0.001) than those with <2 FBs (Table-III).

## DISCUSSION

This study demonstrates that having ≥2 intracranial FBs is strongly correlated with increased mortality in children with PBI. Our findings support previous observations in adults^17^ and suggest that FB count is a valuable prognostic marker in children with high-velocity injuries, such as sniper gunshots and barrel bomb blasts.

Here, we reaffirmed the association between low GCS scores and mortality, demonstrating that a GCS ≤9 significantly predicts poor outcomes. This aligns with previous studies,^18^,^19^ which highlighted that the GCS score at admission is a reliable indicator of survival after PBI.

Moreover, our study identified a strong association between EDH and increased mortality risk, with patients exhibiting an approximately 11.87-fold higher likelihood of death. This is consistent with previous findings,^20^ which showed that traumatic mass lesions such as EDH adversely affect survival rates in PBI cases.

Pre-hospital interventions are crucial in the initial management of patients with PBI. Although PEI is often life-saving, it is associated with poor outcomes, which may be because the need for PEI typically reflects the severity of the initial injury.^21^

Based on our findings, the presence of a midline shift on brain CT was significantly associated with mortality in patients with PBI. Similarly, Puffer et al. reported that among patients with TBI who exhibited midline shift, those with a shift of ≥10 mm had significantly worse prognoses than those without a marked shift at follow-up assessments on days 30, 90, and 180 after admission.^22^

Our subgroup analysis did not reveal significant differences in mortality rates between the sniper and shrapnel groups. However, the shrapnel group showed higher rates of multilobar injuries, ICH, and pneumocephalus. Our findings are consistent with those of Alsalkini et al., who undertook a study examining patients with TBI affected during the SCW^23^ and found no significant difference in mortality rates between cases resulting from shrapnel injuries and gunshot wounds.^23^ A key strength of this study is the relatively large paediatric PBI cohort, collected over seven years during active conflict. Barrel bomb blast injuries often lead to mass-casualty incidents involving multiple paediatric patients. Although the GCS remains a valid predictor of mortality in PBI, in resource-limited settings, CT-based selection of surgical candidates may be more practical for patients with low GCS scores, particularly those with <2 intracranial FBs. To our knowledge, this is the first study to evaluate CT-based prognostic features of paediatric PBI in a civilian population within a conflict zone.

Clinically, the identification of ≥2 intracranial FBs as a prognostic marker is noteworthy. This metric is readily available through initial CT and can help prioritise patients for neurosurgical intervention, especially in resource-constrained environments. Incorporating FB count into neurosurgical candidate selection criteria may optimise clinical decision-making and improve resource allocation.

### Limitations:

This study has limitations, primarily that the patients were followed up for only 24 hours, and information about long-term complications was unavailable. Mortality due to PBI is significantly influenced by various variables, including coagulopathy and hypothermia. However, controlling for all the variables that could influence mortality in PBI cases was not possible. The GCS was evaluated upon initial ED arrival; thus, pre-hospital sedating medications could depress the GCS score along with substances. This study was conducted in a rural hospital lacking an angiography unit. Therefore, detecting potential vascular injuries was not possible. Furthermore, intracranial pressure monitoring and external ventricular drainage could not be performed in the ED.

## CONCLUSION

The number of intracranial FBs can be a prognostic indicator in paediatric patients with PBI. In these patients, the presence of ≥2 intracranial FBs on initial CT is associated with increased mortality. This easily accessible radiological parameter may support early surgical decision-making, especially in resource-limited settings. Further multicentre studies should validate these findings and explore their integration into standardised prognostic models for paediatric neurotrauma in conflict zones.

### Future recommendations:

Future research should validate these findings in multicentre paediatric populations and explore integrating FB count into standardised prognostic models. Additionally, long-term outcome assessments would help evaluate the predictive capability of FB burden beyond initial mortality.

### Authors’ Contributions:

**MB:** Conceptualization, investigation, data collection, supervision.

**KKK:** Methodology, formal analysis, writing—original draft, correspondence.

**YEG:** Methodology development, data curation, writing—review & editing (critical review).

**ACA:** Software, validation, data visualization.

**MB:** Guarantor of the study responsible for the overall integrity and accuracy of the work. All authors reviewed and approved the final version of the manuscript.
